# Stimulation of mouse hematopoietic stem cells by angiogenin and DNA preparations

**DOI:** 10.1590/1414-431X2024e13072

**Published:** 2024-03-04

**Authors:** E.A. Potter, E.V. Dolgova, A.S. Proskurina, V.S. Ruzanova, Y.R. Efremov, S.S. Kirikovich, S.G. Oshikhmina, A.L. Mamaev, O.S. Taranov, A.S. Bryukhovetskiy, L.U. Grivtsova, N.A. Kolchanov, A.A. Ostanin, E.R. Chernykh, S.S. Bogachev

**Affiliations:** 1 Institute of Cytology and Genetics of the Siberian Branch of the Russian Academy of Sciences, Novosibirsk, Russia; 2 Department of Natural Sciences, Novosibirsk National Research State University, Novosibirsk, Russia; 3 LLC “Angiopharm Laboratory”, Novosibirsk, Russia; 4 State Research Center of Virology and Biotechnology “Vector”, Novosibirsk, Russia; 5 Clinical Hospital “Neurovita”, Moscow, Russia; 6 Department of Clinical Immunology, National Medical Research Radiological Centre, Ministry of Health of the Russian Federation, Obninsk, Russia; 7 Research Institute of Fundamental and Clinical Immunology, Novosibirsk, Russia

**Keywords:** Hematopoietic stem cells, Double-stranded DNA, Angiogenin, Multipotent progenitor, DNA internalization, Colony formation

## Abstract

Immature hematopoietic progenitors are a constant source for renewal of hemocyte populations and the basic component of the tissue and cell repair apparatus. A unique property of these cells of internalizing extracellular double-stranded DNA has been previously shown. The leukostimulatory effect demonstrated in our pioneering studies was considered to be due to the feature of this cell. In the present research, we have analyzed the effects of DNA genome reconstructor preparation (DNA^gr^), DNAmix, and human recombinant angiogenin on both hematopoietic stem cells and multipotent progenitors. Treatment with bone marrow cells of experimental mice with these preparations stimulates colony formation by hematopoietic stem cells and proliferation of multipotent descendants. The main lineage responsible for this is the granulocyte-macrophage hematopoietic lineage. Using fluorescent microscopy as well as FACS assay, co-localization of primitive c-Kit- and Sca-1-positive progenitors and the TAMRA-labeled double-stranded DNA has been shown. Human recombinant angiogenin was used as a reference agent. Cells with specific markers were quantified in intact bone marrow and colonies grown in the presence of inducers. Quantitative analysis revealed that a total of 14,000 fragment copies of 500 bp, which is 0.2% of the haploid genome, can be delivered into early progenitors. Extracellular double-stranded DNA fragments stimulated the colony formation in early hematopoietic progenitors from the bone marrow, which assumed their effect on cells in G0. The observed number of Sca1+/c-Kit+ cells in colonies testifies to the possibility of both symmetrical and asymmetrical division of the initial hematopoietic stem cell and its progeny.

## Introduction

Hematopoietic stem cells (HSCs) and their bone marrow stem cell niche comprise a unique cell system that forms and maintains the balance of blood cell elements that repair tissues and organs throughout the life of the organism. The concept of the HSCs is complex. It characterizes a set of cellular states and cell types of different anatomical localizations that differentiate into various cell lineages. There are three classes of HSCs: balanced (Bala), lymphoid-biased (Ly-bi), and myeloid-biased (My-bi), and all of them have different epigenetically fixed differentiation potential. A clonal analysis demonstrated that these cell classes consist of two populations: short-lived HSCs and long-lived progenitors. The first ones differentiate and proliferate within a few weeks, while long-lived progenitors are in the quiescent G0 phase for a long time (1).

It is generally accepted that long-lived dormant HSCs in mice have the Lin-/c-Kit+/Sca-1+/CD150+/CD34-/Flk2-/CD48- phenotype. There is one HSC per 30,000 bone marrow mononuclear cells, and about 80% of HSCs remain quiescent throughout life (in humans) thus maintaining their stemness (2).

A HSC is surrounded by several cell types that form a niche where the HSC functions. The stem cell niche includes endothelial cells, multiple mesenchymal cell types (adipocytes, CXCL12-abundant reticular (adventitial) cells, osteoclast-like cells, leptin R+ and nestin+ cells, and NG2+ arteriolar pericytes), as well as non-myelinating Schwann cells and hematopoietic cells (macrophages and megakaryocytes) (3).

Currently, researchers suggest the existence of two types of HSC niches in adult bone marrow. The osteoblastic niche determines the quiescent state of early primitive progenitors that maintain long-term stemness (LT-HSCs). Upon activation, LT-HSCs differentiate into short-term blood precursors (ST-HSCs) located within the vascular niche adjacent to sinusoidal endothelial cells (4).

The fundamental property of a primitive HSC is its inherent ability to either maintain a quiescent state, divide symmetrically to form two identical HSCs, or divide asymmetrically and give rise to a committed cell with further development of a certain cell lineage.

The potency of HSCs is directly associated with the balance between quiescence and activation. A decreased ability of HSC to exit quiescence leads to insufficient regeneration of blood cells. At the same time, if a disproportionately large number of HSCs exit quiescence or do not return to quiescence after activation, the HSC pool is then depleted, leading to bone marrow failure (5). HSCs of a young organism are known to proliferate and divide symmetrically more often, while progenitors of adult and elderly humans are mostly in a quiescent state (6).

Numerous factors are involved in HSC differentiation. The major factors are anatomic localization of a HSC and the niche maintaining it, as well as the regional hypoxia level. Hypoxia is one of the key factors determining the HSC state, and the majority of quiescent and primitive HSCs are found in hypoxic areas of the brain marrow with low blood perfusion (7). Hypoxic conditions in the HSC are also maintained directly by regulating mitochondrial number and activity. Mitochondria are the main organelles producing reactive oxygen species (ROS). The mitochondrial oxidative phosphorylation is the main source of cellular ROS. Primitive quiescent HSCs with a high potential for stemness were shown to have a low level of ROS. An increase in mitochondrial number and/or activity in HSCs leads to the growth in the number of free radicals in the cell and determines the cell entry into the cycle. The number of mitochondria is regulated by mitophagy, while the quiescent state is due to the abundance of lysosomes, which are organically associated with autophagosomes involved in mitochondrial degradation; inhibition of lysosomes enhances HSC activity by more than 90-fold (8).

Two opposite mechanisms of energy production take place in the HSC under hypoxic and normoxic conditions: oxidative phosphorylation and glycolysis. It is generally accepted that quiescent HSCs use glycolysis to produce energy (9). However, there is evidence that activated HSCs also use glycolysis for energy generation (8).

Functional activity of mitochondria depends on numerous factors. For instance, antioxidant enzymes, reductive peptides, and peroxiredoxins reducing the ROS level are active in HSCs. Stabilization of the HIF-1a/ARNT complex and activation of hypoxia-responsive elements in the genome lead to reduction in mitochondrial oxidative phosphorylation and HSC entry into G0 (10).

Factors secreted by stem cell niches and HSCs, namely membrane-associated factors (4-7,11), are the crucial participants in the processes that form the HSC biological status. As shown for angiogenin, a single factor can act both as an inducer of quiescence for one HSC type and an inducer of cell cycle transition for another cell type (11). Migrating peripheral leukocytes, as well as histamine and tumor necrosis factor (TNF)-α secreted by them and other bone marrow and peripheral blood cells, activate quiescent progenitors (12). Various pharmacological agents, inflammation, hunger, environmental pollutants, and radiation also determine the fate of the HSC (5).

Asymmetric division and subsequent proliferation are the basic mechanisms of regeneration of blood cell populations. This process is a finely regulated chain of events with a diverse and wide range of potent inducers. As discussed above, HSC proliferation and mobilization can be activated by environmental factors and physiological systems of the body acting together as a system of integral stimuli that form a common response vector for HSC and the surrounding microenvironment (stem cell niche). The result of such an effect is activation of molecular signaling cascades and gene sets that determine the fate of the committed daughter cell. Inflammation is one of the factors involved in this process. It is well known that the system of pro-inflammatory cytokines, glucocorticoids (13), and the granulocyte-macrophage (GM) colony-stimulating factor associated with them trigger the entry of quiescent HSCs into the cycle. However, a huge number of self- or pathogen-associated double-stranded DNA (dsDNA) and RNA molecules, both sterile and pathogen-induced, are released into the bloodstream during inflammation (14).

We discovered and described a new general biological property of stem cells of various genesis in our recent studies (15-17). It has been experimentally shown that HSCs, as well as all the analyzed low-differentiated cells of mammals, including tumor-initiating stem cells, are able to capture dsDNA from the environment by a natural mechanism. The interaction of extracellular double-stranded nuclear acids with the cell and its internalization take place with the help of the glycocalyx proteins - proteoglycans/glycoproteins, glycosylphosphoinositol-associated proteins, and the system of scavenger receptors; this process is carried out by a caveolae/clathrin-dependent mechanism. The most important and characteristic feature of the process is the uniqueness of the pattern of proteoglycans/glycoproteins, glycosylphosphoinositol-associated proteins, and scavenger receptor factors on the cell surface of the same type. Furthermore, this uniqueness is limited to three functional protein domains composed of different representatives of these functional domains, namely molecules of the proteoglycans/glycoprotein, glycosylphosphoinositol-associated protein, and scavenger receptor patterns, i.e. each stem cell has three functional protein domains composed of different representatives of these domains, which determine the interaction of the extracellular dsDNA with the cell and its internalization. The main binding site for dsDNA molecules is the heparin-binding domain (the C1q domain, the collagen-binding domain, and positively charged amino acids) found in various cell surface proteins (16,17).

The present study aimed to determine the colony-stimulating potential of two DNA preparations that are commonly used in experimental studies at the Laboratory of Induced Cellular Processes of the Institute of Cytology and Genetics of the Siberian Branch of the Russian Academy of Sciences (LICP of the ICG SB RAS; Russia). Human recombinant angiogenin, which demonstrates a dual effect on early and late progenitors, was selected as a comparison factor (11). The study also quantified early progenitors using specific surface markers c-Kit and Sca-1 and analyzed the ability to capture a TAMRA-labeled dsDNA (as an indicator of dsDNA internalization in poorly differentiated primitive cells) (15). Finally, a quantitative analysis of an extracellular dsDNA internalized in mouse HSCs by real-time PCR was performed.

## Material and Methods

### Animals

Inbred CBA/Lac mice aged 3-5 months were used in all experiments. Mice were provided by the vivarium of the Institute of Cytology and Genetics of the Siberian Branch of the Russian Academy of Sciences. Animals were grown in groups of 6-10 mice per cage. Ssniff food (Germany) and water enriched with the Severyanka (Russia) mineral mixture were provided to animals *ad libitum*. All experiments with animals were conducted in strict compliance with ARRIVE guidelines and were approved by the Animal Care and Use Committee of the Institute of Cytology and Genetics (ICG) SB RAS (protocol No. 48/4 dated 18 March 2019).

### Isolation of bone marrow cells

Mice were euthanized by cervical dislocation. For each experiment, femurs and tibias from 3 mice were dissected and washed with phosphate buffered saline (PBS) (approximately 1-2 mL of PBS per mouse) supplemented with 2% fetal bovine serum (FBS, Capricorn Scientific, Germany). Cells were mixed and thoroughly resuspended by pipetting and strained through a 0.45 μm mesh. Red blood cells were depleted using an erythrocyte lysis buffer (#420301, Biolegend, USA). In short, 1 mL of the buffer was added to the cell pellet and incubated on ice for 5 min with periodic vortexing. Cells were washed once and resuspended in PBS + 2% FBS for cell counting. Approximately 10-15 million cells were isolated from one animal.

### Inducers

Angiogenin was provided by LLC “Angiopharm Laboratory” (Novosibirsk, Russia). The preparation was dissolved in saline at a concentration of 5 μg/mL.

DNA genome reconstructor (DNA^gr®^) was isolated from placentas of healthy women (18). DNA^gr^ was fragmented to 1-20 nucleosomal monomers (∼200-2,000 bp) by sonication, deproteinized using proteinase K, and isolated by phenol-chloroform extraction.

The composite DNA preparation (DNAmix^®^) was a mixture of equal amounts of native human DNA^gr^ and salmon DNA (3:5 ratio of modified crosslinked to native DNA). The sample was prepared as described in a previous study (18).

### Mouse bone marrow cell activation and methylcellulose colony-forming assay

For bone marrow cell activation, the cells were incubated with either angiogenin (300 ng/mL) or DNA preparations (DNA^gr^ and DNAmix, 0.5 mg/10^6^ cells) in Iscove's modified Dulbecco's medium (IMDM) with or without 2% FBS for 2-2.5 h at 37°C, 5% CO_2_, and 95% humidity. For quantification of myeloid progenitor cells, 2×10^4^ bone marrow cells were plated in MethoCult M3434 methylcellulose (Stem Cell Technologies, Canada) according to the manufacturer's instructions. Colonies were counted by visualization on days 7-10.

### Staining of HSC colonies for Sca-1 and c-Kit surface markers and direct assessment of TAMRA-labeled DNA capture on the plates

The amount of anti-mouse Sca-1 FITC (#122505, Biolegend) and c-Kit PE-Cy5 (#105809, Biolegend) antibodies corresponding to the amount required for staining of one test sample according to the manufacturer's protocol (1 µg per million cells) and 0.1 µg of TAMRA-labeled DNA were added to 100 µL of IMDM medium. The resulting solution was spread on plates with HSC colonies without touching the methylcellulose substrate and colonies and then distributed over a small surface area. Data and images were obtained using a Laser scanning confocal microscope LSM 780 NLO and ZenLight software (Zeiss, Germany).

### Evaluation of bone marrow cell staining with surface antibodies for flow cytometry and fluorescence microscopy

Bone marrow cells were resuspended in PBS + 10% FBS and incubated for 10 min at room temperature to block unspecific binding. After this, cells were precipitated by centrifugation at 400 *g* for 5 min at 25°C and resuspended in the final volume of the medium. Cells were aliquoted based on the ratio of 10^6^ cells/0.1 mL of medium for each sample.

For stem and progenitor cell staining, bone marrow cells were stained with antibodies against c-Kit PE-Cy5 (#105809), Sca-1 FITC (#122505), CD34 PE-Cy7 (#119325), Flk2 APC (#135309), a lineage cocktail Lin PE (#133303, 145-2c11/RB6-8C5/RA3-6B2/Ter-119/M1/70), and/or a corresponding isotype control: c-Kit isotype PE-Cy5 (#400609), Sca-1 isotype FITC (#400505), CD34 isotype PE-Cy7 (#400521), and Flk2 isotype APC (#400511); all obtained from Biolegend. Antibodies were added on ice; cells were stained in the dark for 40 min at room temperature. After this, the samples were washed with 0.5 mL of PBS, centrifuged at 400 *g* for 5 min at 4°C and resuspended in 0.25 mL of PBS. Cells were analyzed using a FACSAria III flow cytometer (BD, USA). For microscopy analysis after staining, cells were washed in PBS, resuspended and placed in a 24-well plate. Data and images were obtained using a laser scanning confocal microscope LSM 780 NLO and ZenLight software (Zeiss). For assessment of surface markers in methylcellulose colonies, the recommended amount of each antibody was resuspended in 200 µL of IMDM and added to a Petri dish with colonies.

### Quantification of TAMRA-positive cells in the bone marrow cell population

For quantification of TAMRA+ cells in bone marrow cells and colony cell suspension, 1×10^6^ cells were incubated with 0.1 μg of *Alu*-TAMRA DNA in 400 μL of IMDM in the dark for 30 min at room temperature. After this, cells were precipitated by centrifugation at 400 *g* for 5 min at 25°C, washed with a small volume of medium and resuspended in the final volume of the medium. In the case of c-Kit+/Sca-1+/TAMRA+ cells, staining was performed simultaneously.

### Collection of HSC suspensions of different colony types after methylcellulose assay

After colony quantification, different colony types were carefully harvested from the methylcellulose using pipette tips under a microscope. The cells were then washed from methyl-cellulose with 5 mL of IMDM and centrifuged at 300 *g* for 10 min at 25°C. The resulting cells were washed again with 2 mL of IMDM and resuspended in the medium for quantification.

### TAMRA labeling of DNA probes

Fluorescent labeling of the human *Alu* repeat DNA using polymerase chain reaction-based incorporation of TAMRA-5'-dUTP (deoxyuridine triphosphate) was performed exactly as described (15).

### Incubation of HSC colony cells with the human Alu fragment

The procedure for obtaining HSC colonies is described above. On day 10, colonies obtained after bone marrow cell induction with the DNA^gr^ were collected from two dishes by adding 8 mL of IMDM medium and sedimented by centrifugation at 400 *g* for 8 min. The cells were then washed again with 2 mL of medium and precipitated. The human *Alu* fragment was added to the resulting cells at a concentration of 0.23 μg per 1×10^6^ cells for 30 min at 8 mL of IMDM medium and sedimented by centrifugation at 400 *g* for 8 min. Next, the cells were washed, pelleted by centrifugation at 400 *g* for 5 min, and resuspended in 1 mL of PBS. All procedures were performed at room temperature.

### DNA isolation

MgCl_2_ and DNAse I were added to the cell suspension to the final concentration of 10 mM/mL and 10 µg/mL, respectively. The cells were incubated at 37°C for 15 min, precipitated at 400 *g* for 7 min, and resuspended in H_2_O. Next, the following compounds were added to the cells: EDTA, SDS, and proteinase K to final concentrations of 50 mM, 0.5%, and 50 µg/mL, respectively. The resulting mixture was then incubated at 58°C for 60 min. DNA was purified by phenol-chloroform extraction and re-precipitated by adding 0.1 volume of 3M NaAc and one volume of isopropanol. After centrifugation at 14,000 *g* for 15 min at 4°C, the DNA pellet was dried and dissolved in a small volume of water.

### Quantitative PCR and generation of the calibration curve

DNA molecules were quantified by real-time PCR using a BioMaster RT-qPCR kit (SYBR Green dye) (#RM03-200, Biolabmix, Russia). To generate the quantitative PCR (qPCR) calibration curve, standard M13 primers (M13 for: 5′-GTAAA-ACGAC-GGCCA-G-3′, M13 rev: 5′-CAGGA-AACAG-CTATG-AC-3′) were used, and 0, 0.005, 0.05, 0.5, 5, 50, 500, and 5,000 pg of *Alu* repeat DNA were added to reaction mixtures. Each sample concentration was used in triplicate. The linear fit of Ct *vs Alu* DNA content was plotted using Bio-Rad CFX Manager Software v3.1 (USA). A total of 1, 10, and 100 ng of the DNA from colony cells incubated with exogenous *Alu* DNA fragments were used for quantitative PCR analysis. The concentration of *Alu* fragment in the sample was estimated using Bio-Rad CFX Manager Software v3.1. DNA isolated from control colonies (DNA from bone marrow cells) was used as a negative control (no product whatsoever was observed).

### Conversion of qPCR data into *Alu* DNA copy numbers

Calibration curve-based qPCR data were converted into absolute *Alu* repeat molecule numbers as follows: 100 ng of Krebs-2 DNA added to each qPCR equals to ∼8,333 cells (12 pg/cell). Provided that TAMRA+ cells were shown to be the same cells as those internalizing exogenous *Alu* DNA (both TAMRA-labeled and non-labeled), we could estimate the exact percentage of cells that had internalized the DNA. The fluorescence microscopy analysis showed that 3.5% of cells were external DNA-internalizing (i.e., ∼291 cells). Hence, one can calculate the number of external DNA molecules per cell by dividing the *Alu* copy number measured by 291.

### Statistical methods

Statistical analysis was performed with GraphPad Prism v.8 (GraphPad Software, USA). In the plots, median, interquartile and minimum-maximum range or individual values are shown. Statistical significance was estimated by the Wilcoxon matched-pair test (n=6). Difference was considered significant at P<0.05.

## Results

### The covering note: brief description of the compounds used

The main goal of this study was to estimate the effect of several compounds, which are used in the LICP ICG SB RAS as basic factors for the developing therapeutic approaches (19), on HSCs.

hDNA^gr^ is a DNA preparation that interacts with hematopoietic cells and induces in them a number of processes, which are the subjects of the studies in the laboratory. One of the most mysterious phenomena is the capability of extracellular dsDNA fragments to be natively internalized into the intracellular space of HSCs. It is this phenomenon, in addition to the data obtained in our lab, that was proven in this study using quantitative PCR assay (20,21). This is the same phenomenon we have observed with cancer stem cells (CSCs), which are presumed to utilize the similar molecular machinery for uptaking dsDNA as HSCs do (16). Some data we obtained previously suggest that these internalized dsDNA fragments may be somehow involved in recombination with nuclear DNA with subsequent integration into the genome. We believe that these two phenomena underlie at least two unexplained biological phenomena, namely genometastasis (22) and the considered pseudoscientific hypothesis of telegony. Moreover, such events appear to be the molecular basis for the gene knockout method, which was awarded the Nobel prize in 2007 (https://www.nobelprize.org/prizes/medicine/2007/advanced-information/). The present study is the first report from our lab that describes the initial processes of the interaction of HSCs with extracellular dsDNA. It will be followed by a series of experimental evidence (under preparation for publication) of the possible participation of the internalized dsDNA fragment in the recombination-associated genomic DNA repair processes, which result in the integration of the adscititious genetic information into the recipient genome.

DNAmix, a complex composite dsDNA-based compound, is the main therapeutic agent in the “Karanahan” technology, based on the capability of CSCs to natively internalize extracellular dsDNA fragments (19). It was found that dsDNA fragments that entered into the intracellular space during DNA repair caused by exposure to cyclophosphamide (CP) participate in this process.

In our earlier investigations with bone marrow cells, we demonstrated that delivery of dsDNA fragments to experimental animals during double-strand breaks (DSB; the major intermediates in the repair of CP-induced DNA damage) repair resulted in elimination of the lymphoid lineage, which in turn led to a failure of the immune system with the consequent development of opportunistic infection, systemic inflammation, multiple organ failure, and eventual death of the animal (23).

Further, we proved that such a treatment also ensures the elimination of CSCs, and the underlying element was the capability of CSCs to internalize extracellular dsDNA fragments using a quite natural mechanism described in a previous study (15). The technology aimed to eliminate CSCs was named “Karanahan” or “3+1”, and the main principle underlying this technology is an interference of dsDNA fragments in the repair of CP-induced interstrand crosslinks in CSCs that results in their death or loss of tumorigenicity (19).

DNAmix is the basic therapeutic agent and is to be administered at the time of the two phases of the DNA repair process, non-homologous end joining (NHEJ) and homologous recombination (HR). DNAmix is composed of native human DNA and salmon sperm DNA (both native and crosslinked). The compound components alternately interfere in both phases of the repair process: the first component interferes in NHEJ, while the second one in HR phase. Details of the principle of selecting the compound components as well as their effects on CSCs are described in our previous study (19). It is important to note that DNA repair timing in CSCs and HSCs differs, and “Karanahan” implies that DNAmix administration timing is to be determined for every specific tumor individually. Such an approach allows achieving the maximal therapeutic efficacy against CSCs, while leaving HSCs unaffected. At the moment, the technology implies intratumoral administration of DNAmix that minimizes its effect on distant metastases and dormant CSCs, which are spatially located outside the main tumor node. The issue of intravenous administration of the compound, which would ensure a systemic effect on the entire bulk of CSCs, remains unclear, since should this be the case, DNAmix would reach not only CSCs, but also HSCs. Thus, investigating the effects of DNAmix on HSCs is quite essential for further development and improvement of the “Karanahan” technology.

In this report, we analyzed the effect of DNA^gr^ (a preparation of native human DNA) and DNAmix (a complex DNA compound used in the “Karanahan” technology) on murine HSCs.

Angiogenin was chosen as a comparison factor, since the molecular details of its effect on the cell are known. Angiogenin, also known as RNase5, is a member of the secreted vertebrate-specific Rnase superfamily (24). In growth conditions, angiogenin promotes proliferation and enhances the survival of a variety of cells (25). The growth stimulatory effect of angiogenin is mediated through rRNA transcription (26) and requires nuclear translocation of angiogenin (27). Under stress (generalized cellular stress), angiogenin is translocated to stress granules and mediates the production of tRNA-derived stress-induced small RNA. These small RNA species enhance cell survival by simultaneously suppressing global protein translation, conserving anabolic energy, and permitting protein translation of anti-apoptotic factors mediated by the internal ribosomal entry site (28).

### Quantitative analysis of the colony-stimulating effect of angiogenin, DNA^gr^, and DNAmix inducers

**Figure 1:**
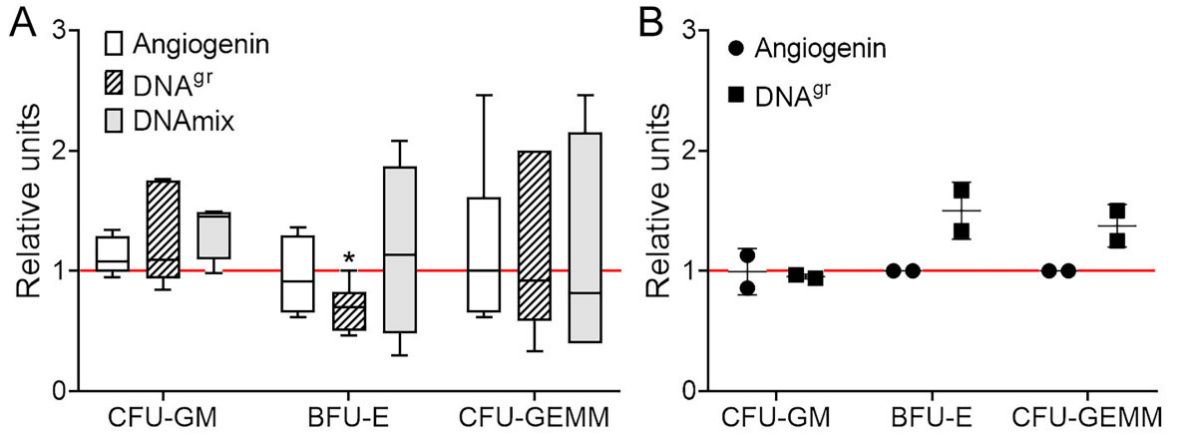
Colony formation analysis. Colony forming units (CFU) of granulocyte-macrophage (GM), burst-forming unit (BFU)-erythroid (E), and CFU of myeloid (CFU-GEMM) hematopoietic precursors by the following inducers: angiogenin, DNA^gr^, and DNAmix, in native conditions (**A**) or in experimentally induced starvation (**B**). Data are reported as median, interquartile and minimum-maximum range (**A**), or individual values (**B**) relative to the CFU of the control colony (red line). *P<0.05 *vs* control group by Wilcoxon matched pairs test (n=6). CFU-GEMM: colony forming unit-granulocyte, erythroid, macrophage, megakaryocyte.

Several experiments were carried out to assess the efficiency of the colony-stimulating action of the selected inducers: angiogenin, DNA^gr^, and DNAmix. All analyzed inducers demonstrated an ability to stimulate the formation of the granulocyte-macrophage hematopoietic lineage (P=0.068). In some cases, the colony number increased 1.7-fold upon induction with DNA^gr^ compared to the control. Inducer DNA^gr^ demonstrated a pronounced tendency to inhibit (P=0.068) formation of the erythroid hematopoietic lineage colonies (Figure 1A).

In addition, colony stimulation in experimentally induced starvation during cell activation was carried out. Cells were incubated for three hours without FBS. No GM lineage activation occurred when using this protocol for samples treated with angiogenin and DNA^gr^. For the erythroid and myeloid hematopoietic lineages, the number of colonies exceeds the control value for the sample treated with DNA^gr^ (Figure 1B).

### Characterization of HSC subsets in intact bone marrow cells of CBA mice

**Figure 2:**
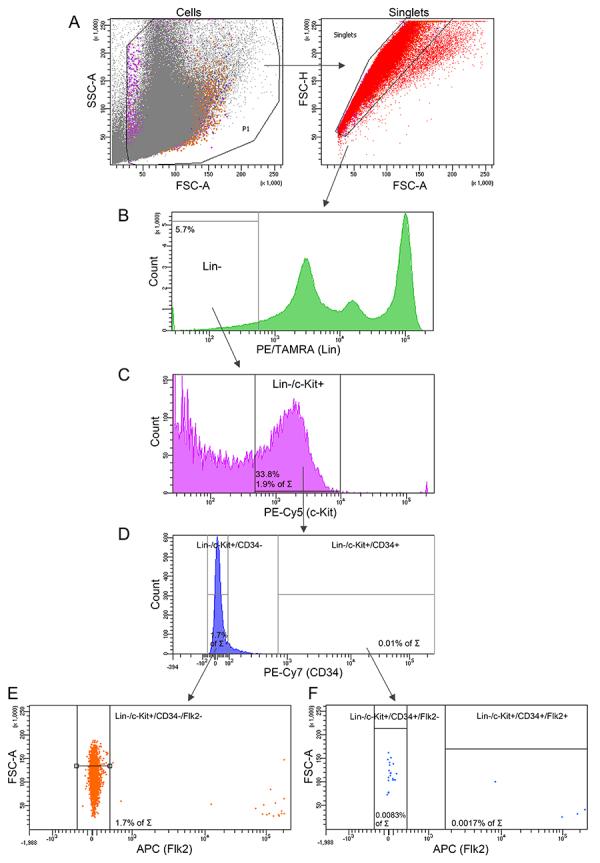
Content of hematopoietic progenitor cell populations in intact murine bone marrow. **A**, Total population of bone marrow cells (left) and singlets isolated from it (right), **B**, Lin- (lineage negative stem cells) population in the total bone marrow cell pool. **C**, Lin-/c-Kit+ (early hematopoietic stem cell (HSCs)) in the Lin- cell population (the percentage of the total bone marrow cells is shown). **D**, CD34+ and CD34- cells in the Lin-/c-Kit+ cell population (data are presented as a percentage of the total bone marrow cell pool). **E**, Number of Lin-/c-Kit+/CD34-/Flk2- (long-term HSCs, LT-HSCs) cells in the Lin-/c-Kit+/CD34-population found in C. **F**, Number of Flk2+ (multipotent progenitors) and Flk2- (short-term HSCs, ST-HSCs) populations in Lin-/c-Kit+/CD34+ bone marrow cells.

Intact bone marrow cells of CBA mice were assessed for HSC surface markers by flow cytometry analysis (Figure 2, Table 1). The content of cells with stemness markers was in accordance with other studies in the mouse model (29). The complete phenotype of HSCs (Lin-/c-Kit+/Sca-1+/CD34-) was not found in the analysis because the Sca-1+ marker was not detected in combination with other markers. For this reason, the Lin-/c-Kit+/CD34- population was further analyzed as HSCs.

**Table 1:** Surface markers of intact bone marrow cells of mice analyzed by flow cytometry.

Type of cells	Surface markers	Content of total population (%)
Early HSCs	Lin-/c-Kit+	1.9
HSCs	Lin-/c-Kit+/CD34-	1.7
LT-HSC	Lin-/c-Kit+/CD34-/Flk2-	1.7
ST-HSC	Lin-/c-Kit+/CD34+/Flk2-	0.0083
Multipotent progenitors	Lin-/c-Kit+/CD34+/Flk2+	0.0017

HSC: hematopoietic stem cell; LT: long-term stemness; ST: short-term stemness.

### Stimulation of colony formation with recombinant human angiogenin, DNA^gr^, and DNAmix

**Figure 3:**
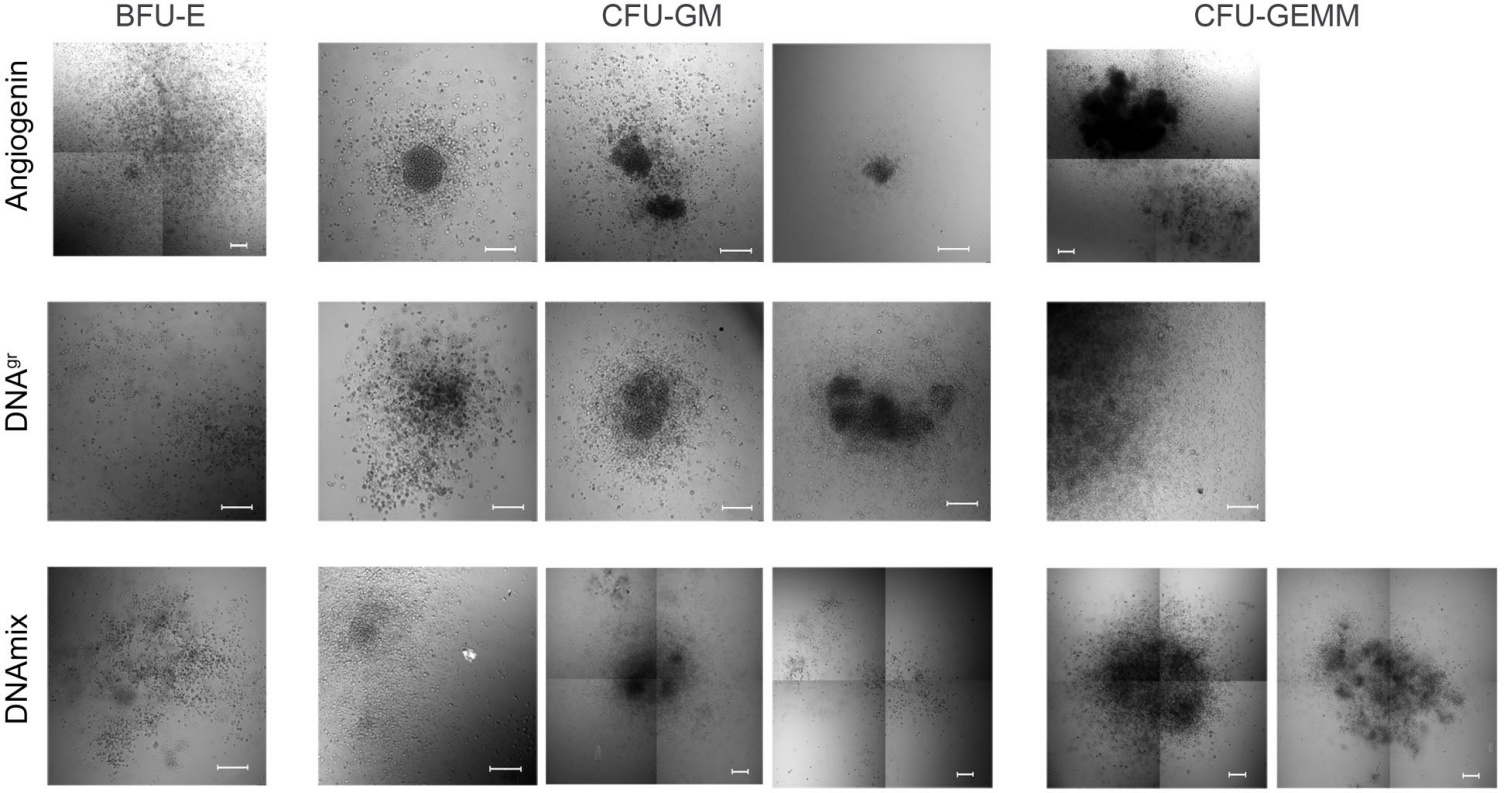
Morphology of colonies on day 10 after bone marrow cell stimulation with angiogenin, DNA^gr^, and DNAmix. BFU-E: burst forming unit-erythroid; CFU-GM: colony-forming units-granulocyte-macrophage; CFU-GEMM: colony forming unit-granulocyte, erythroid, macrophage, megakaryocyte. Scale bar, 200 µm.

Bone marrow cells isolated from the mouse femur were stimulated with either angiogenin DNA^gr^ or DNAmix for 2.5 h under standard conditions in a CO_2_ incubator, washed from the inducer, and seeded onto a methylcellulose substrate according to the commercial protocol. Figure 3 shows the characteristic morphology of the three main colony types.

### Primary analysis of the possibility of co-localization of primitive HSC markers c-Kit and Sca-1 and ability of these cells to internalize TAMRA-labeled dsDNA in colonies grown on plates using various inducers

This study is the first step in creating a new technology for HSC genome reconstruction/equalization based on the discovered ability of poorly differentiated cells, including HSCs, to capture dsDNA by a natural mechanism described in previous studies (16,17). The proposed analysis is the basis for the technological platform for obtaining a primary *in situ* screenshot of the colony phenotype and genetic changes directly on plates. Also, the analysis made it possible to link three marker factors and link them to the type of colony.

After induction using the above-mentioned preparations in methylcellulose dishes, the colonies were stained for c-Kit and Sca-1 markers, and the ability of these cells to capture the TAMRA-dsDNA was determined. The following trends were noted.

#### Recombinant human angiogenin

**Figure 4:**
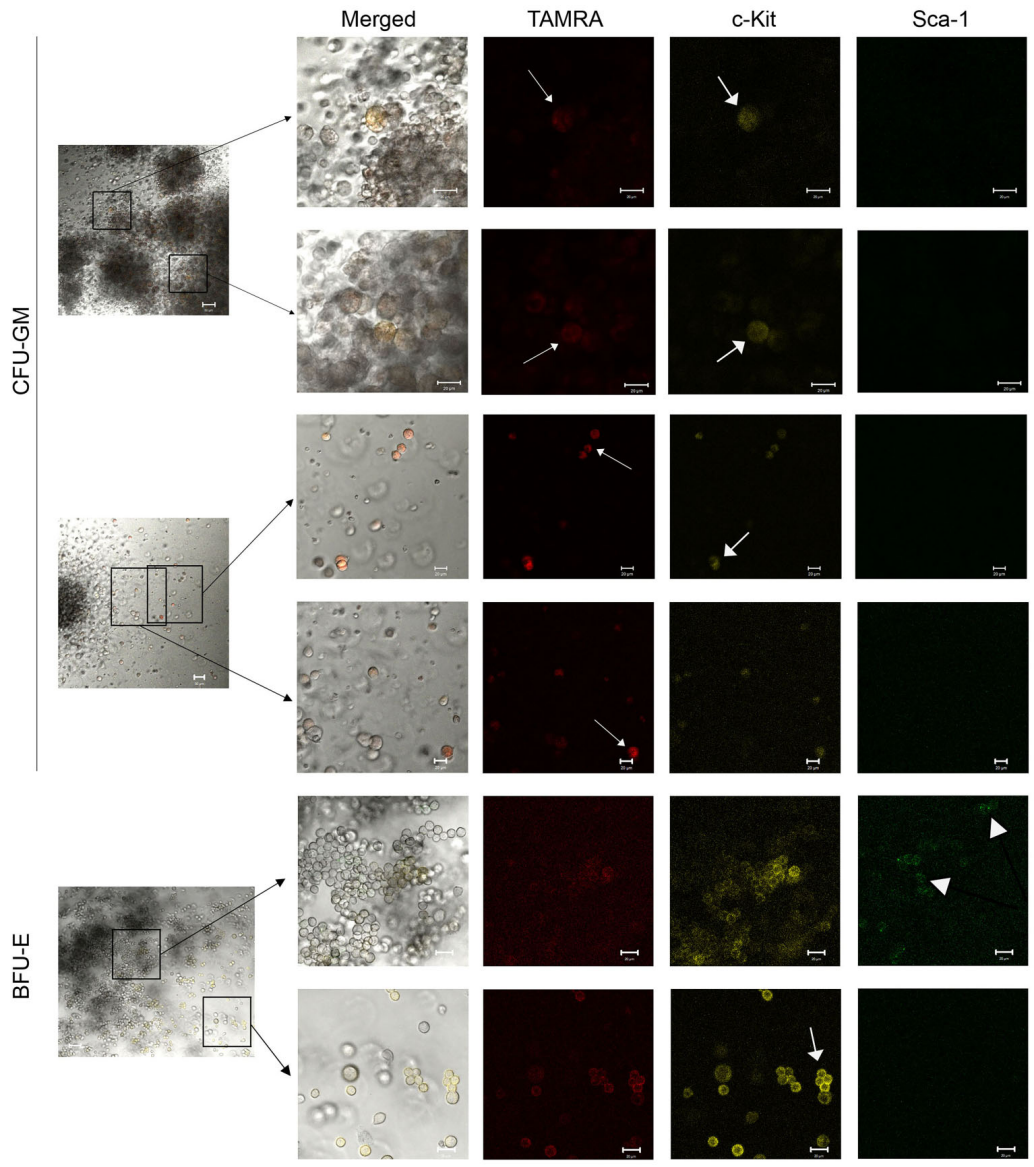
Analysis of the TAMRA-labeled dsDNA internalization into hematopoietic stem cells (HSCs). Arrows indicate the cells internalizing TAMRA-labeled dsDNA probe or marked with antibodies fluorescent signal. Representative staining trends in different cell colonies (BFU-E and CFU-GM) with Sca-1 and c-Kit surface markers of hematopoietic precursors and uptake of TAMRA-labeled DNA by cells of these colonies after bone marrow cell activation by angiogenin. Scale bar, 20 µm. BFU-E: burst forming unit-erythroid; CFU-GM: colony-forming units-granulocyte-macrophage; dsDNA: double-stranded DNA.

Colony forming units (CFU)-GM: many cells were stained with the TAMRA marker, with about 50% of them being stained with c-Kit. No cells positive for Sca-1 were found. TAMRA+/c-Kit+ cell clusters were found at the colony periphery, with all c-Kit+ cells being TAMRA+, but not vice-versa (Figure 4). Burst-forming unit (BFU)-erythrocyte (E): almost all cells were stained for c-Kit, especially at the colony periphery, where the cells were located more loosely. The number of c-Kit+ cells exceeded the number of TAMRA+ cells. Sca-1+ cells were present, and almost all of them were positive for c-Kit. However, the intensity of staining for c-Kit of Sca-1+ cells was weak. Only a few individual Sca-1+ cells were stained for TAMRA (Figure 4).

Preliminary conclusions were drawn based on the analysis results. The Sca-1 marker was characteristic of erythroid cells and megakaryocytes (data not provided) and absent in macrophage (data not provided) and granulocyte colonies. Cells carrying the Sca-1 marker often overlapped with c-Kit+ cells and rarely overlapped with TAMRA+ cells. The erythroid lineage cells were intensely stained for c-Kit, and this staining mainly coincided with cells capturing the TAMRA-labeled DNA, but the number of the latter cells was lower. Granulocyte-macrophage colonies were characterized by double TAMRA+/c-Kit+ staining, but only TAMRA+ cells predominated.

#### DNA^gr^

**Figure 5:**
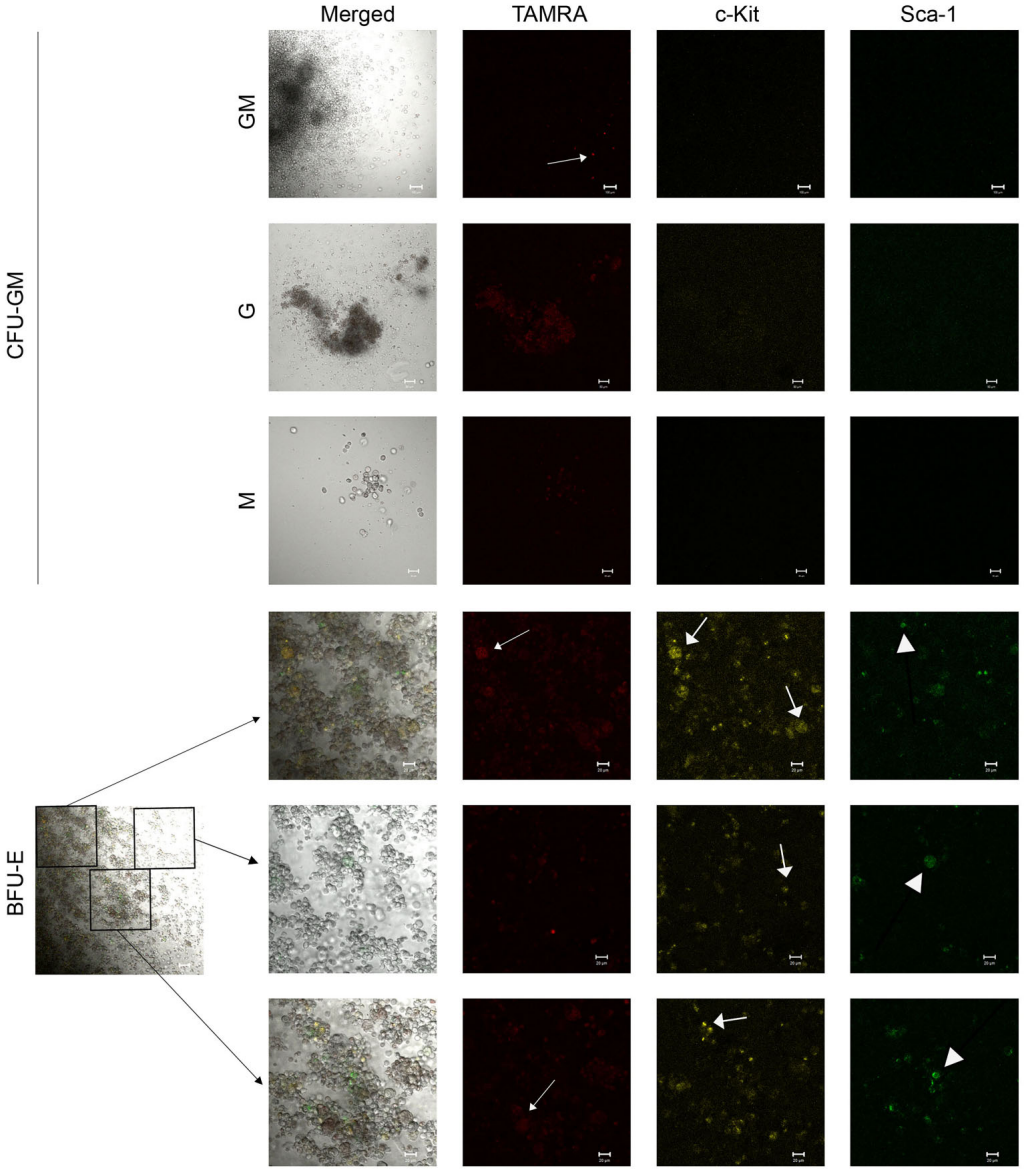
Analysis of the TAMRA-labeled dsDNA internalization into hematopoietic stem cells (HSCs). Arrows indicate the cells internalizing TAMRA-labeled dsDNA probe or marked with antibodies fluorescent signal. Representative staining trends in different cell colonies (BFU-E and CFU-GM) with Sca-1 and c-Kit surface markers of hematopoietic precursors and uptake of TAMRA-labeled DNA by cells of these colonies after bone marrow cell activation by DNA^gr^. Scale bar, 20 µm. BFU-E: burst forming unit-erythroid, CFU-GM: colony-forming units-granulocyte-macrophage; dsDNA: double-stranded DNA.

CFU-GM: the cells of these colonies almost did not stain for c-Kit and Sca-1. The cells were stained for TAMRA in looser colonies in the mass and predominantly along the periphery in colonies with a dense core (Figure 5). BFU-E: similar to the colonies stimulated by angiogenin, a large number of cells were stained for c-Kit, a few cells were stained for Sca-1, and there were also cells that were stained simultaneously for both markers. There were fewer TAMRA+ cells than c-Kit+ cells; TAMRA+/c-Kit+ and TAMRA+/Sca-1+ cells, as well as single cells positive for all three markers, were observed (Figure 5).

In general, we can say that similar to the angiogenin-induced activation, there were almost no Sca-1+ cells in CFU-GM colonies after activation by DNA^gr^. Erythroid lineage cells were similarly characterized by intense staining for c-Kit and staining of individual cells for the Sca-1 marker. The number of TAMRA+ cells in these colonies was lower than that of c-Kit+ cells, with some cells having double TAMRA+/c-Kit+ staining, while Sca-1+ cells rarely expressed only one of the markers.

#### DNAmix

**Figure 6:**
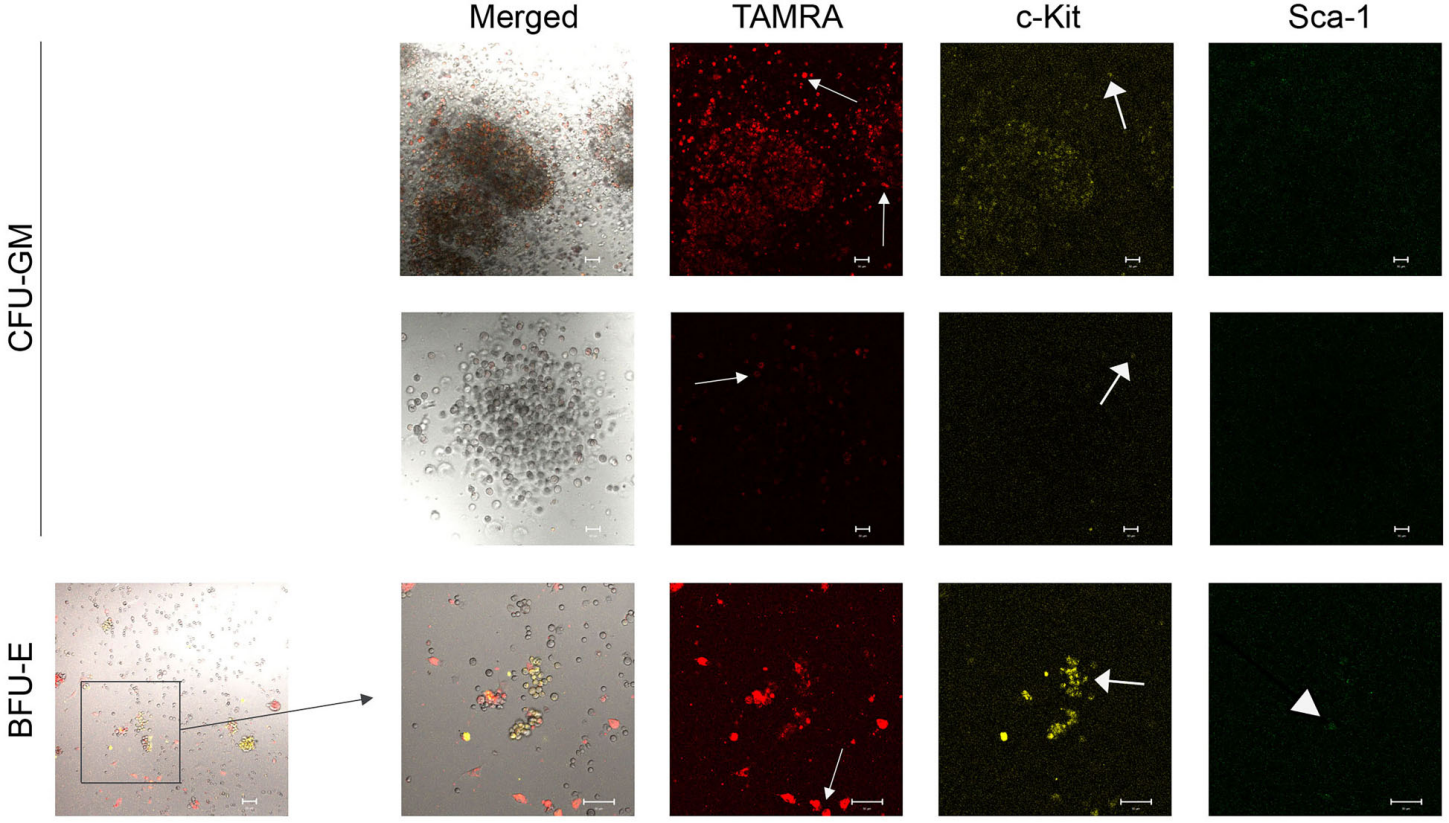
Analysis of the TAMRA-labeled dsDNA internalization into HSCs. Arrows indicate cells internalizing TAMRA-labeled dsDNA probe or marked with antibodies fluorescent signal. Representative staining trends in different cell colonies (BFU-E and CFU-GM) with Sca-1 and c-Kit surface markers of hematopoietic precursors and uptake of TAMRA-labeled DNA by cells of these colonies after bone marrow cell activation by DNAmix. Scale bar, 20 µm. BFU-E: burst forming unit-erythroid; CFU-GM: colony-forming units-granulocyte-macrophage; dsDNA: double-stranded DNA.

CFU-GM: numerous TAMRA+ cells, some of them were also stained for c-Kit, were observed, but the staining was not intense. No Sca-1+ cells were detected (Figure 6). BFU-E: there were very few Sca-1+ cells; their staining intensity was weak but specific. Almost all Sca-1+ cells had the c-Kit+ marker; numerous c-Kit+ cells, as well as TAMRA+ cells, were found, most of them overlapped and expressed the double TAMRA+/c-Kit+ marker (Figure 6).

The main trends in colony staining for the studied markers upon DNAmix activation corresponded to those of the angiogenin and DNA^gr^ groups.

Direct analysis of colony staining in the methylcellulose dish for three stemness markers (TAMRA-labeled DNA capture, and c-Kit and Sca-1 surface markers) revealed the following trends for all three inducers. The vast majority of cells in granulocyte-macrophage colonies were stained for TAMRA, with some of them also expressing the c-Kit+ marker. No Sca-1+ cells were observed. The cells were stained more intensely at the colony periphery than in the mass. Erythroid colonies were characterized by intense staining of most cells for c-Kit. Part of these cells captured TAMRA-labeled DNA (TAMRA+). Sca-1+ cells were also present, many of them stained for c-Kit, and some of the cells were stained for TAMRA+. Cells with triple TAMRA+/c-Kit+/Sca-1 staining were rare.

Thus, we found that different hematopoietic lineages were characterized by different patterns of staining for stemness markers during their differentiation. The erythroid lineage had predominantly c-Kit+ cells, single TAMRA+, and Sca-1+ cells, and the granulocyte-macrophage lineage had the majority of the cells TAMRA+; little c-Kit+ and no Sca-1 were found.

The described property of the cells of different colonies can be used as an additional feature in determining the colony type based on its morphology.

### Analysis of co-localization of primitive HSC markers c-Kit and Sca-1 and ability of these cells to internalize TAMRA-labeled dsDNA in intact bone marrow cells and purified cells isolated from colonies obtained after activation with different inducers (quantitative characterization)

**Figure 7:**
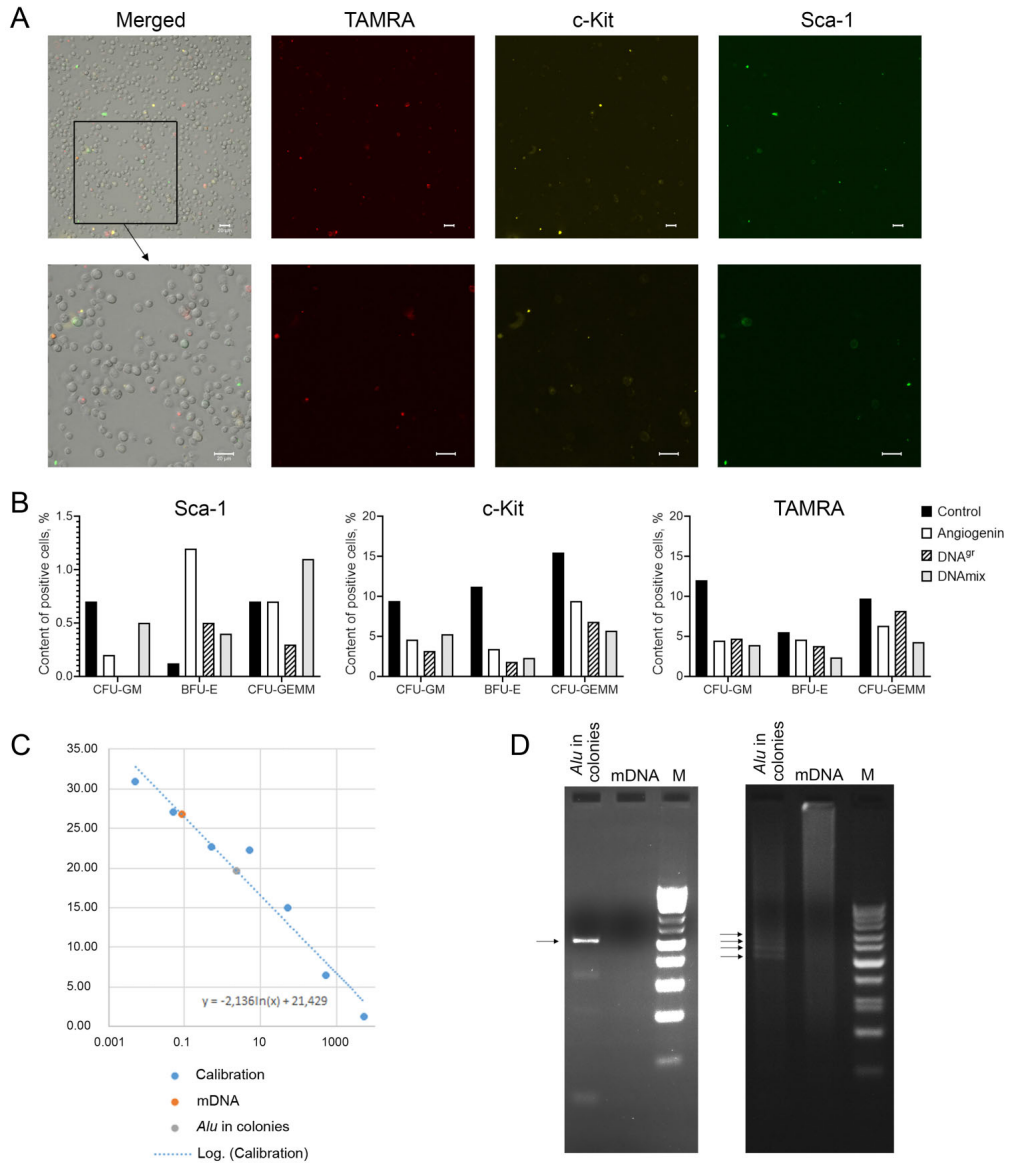
**A**, Simultaneous evaluation of co-localization of primitive hematopoietic stem cell (HSC) markers c-Kit and Sca-1 and ability of these cells to internalize the TAMRA-dsDNA in intact murine bone marrow. Scale bar, 20 µm. **B**, Comparative graphs of the content of cells (%) labeled with the corresponding marker in individual cell colonies. An increase in the Sca-1 level in erythroid (BFU-E) colonies indicates that the Sca-1 cells started to divide symmetrically in this lineage, and the number of HSCs in the colony increased. A decrease in the Sca-1 level in CFU-GM colonies indicates that Sca-1 cells in this lineage passed the point of commitment upon induction. A decrease in the c-Kit level in BFU-E and CFU-GM colonies shows that c-Kit cells became committed upon induction. A decrease in the number of TAMRA+ cells in BFU-E and CFU-GM colonies shows that, similar to c-Kit cells, stem cells became committed upon TAMRA+ induction. **C**, Quantification of the copies of internalized dsDNA. The calculation shows that the dsDNA level is ∼14,000 copies per cell. **D**, Electrophoretic mobility of the PCR fragment obtained using M13 primers and the DNA template isolated from mouse HSCs induced with DNA^gr^ and incubated with the human *Alu* fragment. On the left: the PCR product from templates “*Alu* in colonies” and mouse DNA. On the right: electrophoresis of RT-PCR products from similar templates. Arrows indicate PCR products synthesized from the concatameric dsDNA template *Alu.* BFU-E: burst forming unit-erythroid; CFU-GM: colony-forming units-granulocyte-macrophage; CFU-GEMM: colony forming unit-granulocyte, erythroid, macrophage, megakaryocyte; dsDNA: double-stranded DNA.

We first analyzed co-localization of primitive HSC markers c-Kit and Sca-1 and the ability of these cells to internalize the TAMRA-dsDNA in intact bone marrow cells of CBA mice. A total of 10^6^ bone marrow cells were stained for surface markers c-Kit and Sca-1, and the TAMRA-labeled dsDNA was added to them, followed by 30-min incubation. After that, the cells were washed and analyzed under the microscope to count stained cells (Figure 7A and B).

We found that the number of c-Kit-expressing cells prevailed and accounted for 9.4%. There were 5.4% TAMRA cells and 1.7% Sca-1+ cells. Content of cells with double TAMRA+/c-Kit+ staining was 1.3%. Single cells with the TAMRA+/Sca-1+ and Sca-1+/c-Kit+ phenotypes (0.39% each) and several cells with the triple TAMRA+/Sca-1+/c-Kit+ phenotype (0.35%) were found.

In order to characterize the cells of individual colonies, bone marrow cells were stimulated with either angiogenin, DNA^gr^, or DNAmix in a CO_2_ incubator for 2.5 h under standard conditions, washed from the inducer, and seeded onto a methylcellulose substrate according to the commercial protocol. On day 8, colonies of each type were individually selected from the dishes, washed from methylcellulose, and stained for TAMRA, c-Kit, and Sca-1 markers. We intended to estimate the content of cells expressing each of these markers and their combinations in different colony types. The number of cells stained for each marker was evaluated both by microscopy and flow cytometry (Figure 7B, flow cytometry data not shown). The results obtained during cell counting showed a similar pattern in the percentage of cells stained for each of the studied markers.

Analysis of cell staining in suspension revealed that the content of Sca-1+ cells in granulocyte-macrophage colonies was extremely low and amounted to 0.7% in the control group, 0.2% in the angiogenin group, 0% in the DNA^gr^ group, and 0.5% in the DNAmix group. These data were consistent with evaluation of the content of Sca-1+ cells directly in the colonies in methylcellulose: no such cells were detected in any experimental group. Firstly, the presence of single Sca-1+ cells can be explained by contamination with cells of other colony types during colony sampling from methylcellulose. Secondly, it can also be due to the fact that, during *in situ* analysis, these cells were located in the center of colonies and could not be detected or stained in the cell mass.

The content of TAMRA+ cells was quite high in all types of colonies and for all inducers, as well as in the control; it ranged from 2.4 to 12% and was highest in colonies of the control group. The number of c-Kit+ cells was also high and ranged from 1.8 to 15.5%; its maximum was also observed in the control group. We analyzed the data obtained from two colony types: BFU-E and CFU-GM. The following pattern of the content of cells with the studied markers in induced samples was observed compared to the control (Figure 7B).

#### TAMRA marker

A pronounced decrease in the percentage of cells capable of capturing extracellular dsDNA was observed in both colony types and induced by all inducers.

#### c-Kit marker

A significant decrease in the percentage of cells with the c-Kit phenotype was found in erythroid colonies induced by all inducers. The percentage of cells with the c-Kit phenotype was reduced in GM colonies, although not significantly.

#### Sca-1 marker

A significant decrease in the percentage of cells with the Sca-1 phenotype was noted in erythroid colonies activated by all inducers. The percentage of cells with the Sca-1 phenotype in granulocyte-macrophage colonies did not differ significantly from that of the control.

### Real-time PCR analysis

The present study, as well as our earlier studies (21), showed that CD34+ Sca-1/c-Kit HSCs actively internalize the TAMRA-dsDNA. The internalization mechanism and factors are described in detail in previous studies (16,17). In order to once again demonstrate the internalization of extracellular fragments by HSCs, the following experimental approach was applied. The pooled cell population consisting of all colonies from the plate induced by DNA^gr^ was incubated with the *AluI* dsDNA (a complete analogue of the TAMRA-dsDNA probe without the fluorochrome) according to the procedure described in the Material and Methods section. After the cells were washed from the substrate, they were exhaustively treated with DNase I and then PrK to completely eliminate the external dsDNA. The procedure was carried out according to the protocol described in a previous study (16). Previously, all washings and supernatants of cells treated with the dsDNA had been repeatedly analyzed in order to detect residual dsDNA molecules. Repeated PCR analysis demonstrated the complete absence of extracellular template. The use of the above-mentioned treatment protocol made it possible to avoid using numerous additional PCR controls in experiments.

Total DNA was isolated from the cells treated as described above. Total DNA isolated from bone marrow cells was used as the control. The real-time PCR results are presented in Figure 7C. The calculation indicated that ∼14,000 copies of the ∼500 bp fragment, which comprises ∼6.5×10^6^ bp and 0.2% of the haploid genome, were delivered to the cell at the selected dsDNA probe/cell number ratio (the point of reaching the internalization plateau). The previously obtained results that extracellular fragments either self-ligate or form covalently bound concatemers upon entering inner cell compartments were confirmed (21) (Figure 7D).

## Discussion

The first conclusion of this study was that double-stranded nuclear acid fragments (hDNA^gr^, DNAmix) can induce maturation/differentiation of primitive hematopoietic precursors. This process takes place either upon interaction between the inducer and HSC cytoplasmic membrane factors or upon entry of double-stranded nuclear acid fragments into the progenitor cell's inner space. The granulocyte-macrophage hematopoietic lineage responds to the stimulus most robustly. A comparative analysis of stimulation with the selected inducers is presented in the Results section of the study.

Numerous factors stimulating HSC differentiation are known (30). Inflammation is one of the body conditions in which many factors are released into the area surrounding the inflammatory lesion, which is a necessary and inevitable consequence of the process. These factors are various cytokines, growth factors, and prostaglandins, which trigger stem cell differentiation and proliferation when entering the bone marrow (31). Both LT-HSCs and ST-HSCs can be involved in this process (4,32).

It is well known that tissue injury during inflammation results in occurrence of self- and pathogen-derived nuclear acids in the bloodstream, lymph, and interstitial fluids at levels exceeding the normal range (33,34). This nucleosomal DNA induces necrosis of lymphocytes, activates pro-inflammatory responses of immune cells, and forms a feedback loop that enhances the inflammatory process, leading to a systemic inflammatory response and multiple organ failure.

Unfortunately, during literature analysis, we found little data regarding both the involvement of plasma DNA in HSC activation and the overall effect of this trigger on primitive bone marrow cells in the form of a direct effect of DNA molecules. The mediated effect of pathogen and self DNA on HSCs through the system of immune cells and their secretion of pro-inflammatory cytokines, which in turn interact with HSCs, has been reported (31).

Recombinant angiogenin was shown to inhibit maturation of primitive HSCs by maintaining and preserving their stemness while stimulating proliferation of myeloid restricted progenitors. Angiogenin mediates tRNA-derived stress-induced small RNA production in HSCs and promotes rRNA transcription in myeloid-restricted progenitors. This effect of angiogenin is apparently associated with the metabolic state of these cells. In primitive HSCs, which are under hypoxic conditions (an element of generalized cellular stress), angiogenin translocates to the cytoplasm and activates the synthesis of anti-apoptotic genes, suppresses global protein translation, and conserves anabolic energy. In myeloid restricted progenitor cells that have left G0 and hypoxic stress, angiogenin is preserved in the nucleus and stimulates rRNA transcription and cell growth. These properties of angiogenin are reflected by enhanced hematopoietic regeneration and animal survival upon treatment with recombinant angiogenin protein following radiation-induced bone marrow failure and a dramatic increase in the level of hematopoietic reconstitution by angiogenin-treated mouse LT-HSCs (long-term HSCs) and human CD34+ cord blood cells (11,35).

The second conclusion of the study was the acknowledgement of the fact that dsDNA is captured by HSCs. The interaction between extracellular fragments and HSCs stimulates differentiation of the latter. No mechanism of action that can define the progenitor fate has been described for dsDNA molecules entering a cell. One of the most important questions related to dsDNA internalization in HSCs is whether a dsDNA can induce the activation of primitive totipotent cells in the G0 phase and what could be a possible mechanism for launching this process.

The current study showed that Sca-1+, c-Kit+, and c-Kit+/Sca-1+ HSCs internalize the TAMRA-labeled dsDNA. Approximately 14,000 copies of the 500-bp dsDNA are delivered to HSC internal compartments, meaning that the dsDNA is delivered to cells with markers characterizing primitive HSCs, which, according to the existing concept, are in the G0 phase of the cell cycle (there are cells with different sets of markers, ranging from one to all three markers per cell among HSC descendants in the colonies).

It has also been found that cell treatment with dsDNA induces maturation of HSCs and proliferation of differentiated progenitors. A change in the number of colonies in the samples demonstrates either an increase in the number of a certain type of colonies by 65% or a decrease by 40% compared to the control, depending on the experiment and the inducer used.

The studies performed by us (20), other researchers (36,37), as well as the present study, show that extracellular DNA fragments are processed and ligated into either a circle or a concatemer (this form is not a stimulus and can be present inside the cell for a long time). This means that the reparative mechanism is activated upon dsDNA internalization. The ends of dsDNA are the strongest molecular stimulus and a trigger of checkpoint events that arrest the cell cycle. In general terms, the mechanism is as follows: the occurrence of a double-strand break in the cell first leads to ataxia-telangiectasia mutated kinase (ATM) activation, which then phosphorylates its own substrates (kinases), including DNA-PrK, which is involved in DSB repair, as well as factors responsible for cell cycle arrest (Chk1, Chk2, and P53).

The induction of cell cycle arrest is determined by phosphorylation of Chk1 and Chk2, which in turn hyperphosphorylates the CDC25 phosphatase and thus inactivates it. In normal conditions, CDC25 dephosphorylates the cyclin/cyclin-dependent kinase complex (CDK) thus maintaining its functional state. CDK phosphorylation inhibits the transfer of CDC45 to the origin of DNA replication, and the synthesis stops (38). Thus, CDC25 regulates cell cycle progression through CDK activation, which, in addition to the Rb/E2F complex activation, is also involved in activation of the MCM2-7 hexamer (DNA helicase). This hexamer is part of the pre-replication complex; it catalyzes helix melting during replication.

The repair is initiated after cell cycle arrest. One can assume that, regardless of the stimulus (oxidative stress or gamma radiation (39)), ATM activates the global mechanisms of repair and restoration of molecular order in the cell. We suggest that, after the repair is completed, dephosphorylation of the CDC25 phosphatase takes place, which is followed by dephosphorylation of the cyclin-dependent kinase complex, which inevitably leads to the obligatory passage through the cell cycle of HSCs that had previously been in G0 (40).

This result suggests that the dsDNA entry into primitive cells triggers a proliferative shift and subsequent division among a certain percentage of these cells. If the cells had not been primitive but already multipotent at various differentiation stages, they would have provided colonies in any case and in the same number as in control samples. We would observe an increase in the number of colonies. An alternative hypothesis suggests that, if pluripotent progenitors (which still express totipotency markers but are no longer totipotent) after the first division are in G0 for a certain time, then, like totipotent primitive cells, their division will be induced by dsDNA, and they will form colonies in methylcellulose.

The characteristic presence of cells stained with all three markers in the colonies indicates the possibility of both symmetric and asymmetric division of the initial totipotent HSC and its descendants. Evaluation of the number of Sca-1 and c-Kit cells demonstrates the following: Sca-1+ cells divide symmetrically, which is indicated by an increase in the cell number in colonies expressing this marker after stimulation; c-Kit+ cells become committed, which is manifested as a significant decrease in the number of cells in the colonies.

The question about the functional load of the mechanism of extracellular dsDNA uptake remains open. There are different suggestions about it. Firstly, as it has been discussed, it can be a response to inflammation. The proliferation of immune system cells, which are aimed to cease the inflammatory process, is induced. It is possible that this is one of the ways for obtaining a “nutrient” substrate in the form of the nuclear acid. An exotic alternative is also possible. The transient presence of self DNA fragments in HSCs (20) is a molecular mechanism of detecting changes occurring in the genome, to which HSCs can respond with reparative recombination to “attention-grabbing” dsDNA with either altered sequences or epigenetic modifications.

The described phenomenon of internalization of extracellular dsDNA and whole plasmids (21) by HSCs creates a new paradigm of *in vivo* genome reconstruction/equalization in the form of a single nucleotide polymorphism (SNP) or allelic correction of modified chromosomic DNA regions leading to the development of a pathology by either targeted or genome-wide stochastic transient extracellular therapeutic dsDNA. Within the new paradigm, the approaches of targeted genome correction using complex-targeted biological constructs (TALEN and CRISPR/Cas9) and those related to the creation of conditions for intrinsic, naturally occurring, stochastic, or targeted homologous recombination that either randomly or directionally restore a mutant SNP or allele (the recombination event (40)) with the help of extracellular fragments have the same initial development potential.
